# Impact of the Regulatory Framework on Medical Device Software Manufacturers: Are the Guidance Documents Supporting the Practical Implementation? Comment on "Clinical Decision Support and New Regulatory Frameworks for Medical Devices: Are We Ready for It? – A Viewpoint Paper"

**DOI:** 10.34172/ijhpm.2023.7470

**Published:** 2023-07-10

**Authors:** Ruth Beckers, Pascale Van Hoydonck

**Affiliations:** Qualix BV, Leuven, Belgium

**Keywords:** Clinical Decision Support, Software, Practical Implementation, International Guidance Documents, EU, US

## Abstract

The increasing use in clinical practice of software such as mobile apps and clinical decision support (CDS) software has only recently been taken up by regulators around the world. Specifically, the European Commission and the US Food and Drug Administration (FDA) have updated their regulatory framework in the last years. Van Laere et al have given an extensive overview of the European and US approaches to regulate CDS software. This commentary further discusses regulatory differences between the two geographies and their impact on manufacturers of medical device software. We discuss the practical implementation of the regulatory framework for medical device software (especially CDS software) with a reference to the available international guidance documents and their limitations. Given the direction of stricter regulatory oversight in Europe, additional European guidelines/examples are desirable to enable a pragmatic regulatory approach ensuring continued access to innovative medical device software for European patients.

## Introduction

 Just as other sectors, the healthcare sector has recently experienced an increased digitalisation. This tendency caused the widespread use of software, for example to streamline administrative processes within hospitals and care centres (information technology equipment), but also to support clinical decision making by healthcare professionals (HCPs). Van Laere et al^1^ explain the different regulatory frameworks that apply to clinical decision support (CDS) software in the United States (Food and Drug Administration, FDA, 21st Century Cures Act)^2^ and in Europe (European Union Medical Device Regulation [MDR]).^3^

 We will mainly comment and ask critical questions on the practical implementation of the regulatory framework for medical device software (especially CDS software), with a reference to the available international guidance documents and their limitations. As mentioned by Van Laere et al,^1^ manufacturers of medical device software need to consider those regulatory frameworks carefully as they might impact their time and costs to market significantly.

###  Importance of Keeping Guidelines Up to Date With the “State of the Art” Software 

 There is a general trend of digitalization in healthcare with a broad spectrum of different medical and non-medical device software functions used. This is confirmed by policy-makers through providing illustrative examples in guidance documents for the qualification of software used in the healthcare environment.^4-10^ It is understood that the examples in the guidelines have been drafted in the light of today’s state of the art. However, we hope that those guidance documents will be treated by policy-makers as living documents that will be updated with innovative examples (eg, software algorithms that operate via machine learning or other artificial intelligence [AI] techniques) following the pace of evolving technologies. It is correctly noted in the viewpoint article that there is an assumption that EU MDR will hamper software development as the FDA Guidances^4-9^ present more innovative software examples than the EU Medical Device Coordination Group (MDCG) Guidance Documents.^10^

###  Qualifying Software as a Medical Device – Is the Definition Clear?

 To be qualified as a medical device, a product must first fulfil the definition of a medical device according to the applicable legislation. The international harmonization body (former Global Harmonization Task Force or International Medical Device Regulators Forum [IMDRF]) has created non-binding guidance documents (eg, definition of the term “Medical Device”) to encourage regulatory systems’ convergence at the global level by eliminating differences between jurisdictions. Harmonized guidance would ideally result in decreasing the cost of gaining regulatory compliance and in allowing patients earlier access to innovative technologies and treatments.^11^ The EU MDR definition and its specification of the medical purpose of a device is more leaning towards the Global Harmonization Task Force definition compared to the device definition in section 201 (h) of the Food, Drug & Cosmetic Act.^2^ In addition, the term “monitoring” in the EU MDR^3^ is focused on products intended to monitor physiological processes, while “monitoring” is not present in the FDA device definition.^2^ Unfortunately, there is no clear definition of the term “to monitor” which complicates the qualification of a software product. We would suggest defining monitoring as *“following the evolution of a disease, injury/disability or physiological or pathological process or state at different stages or at different moments in time.”* Many software products are intended to follow up chronic patients at home by visualising parameters measured with different hardware medical devices and notifying deviations to enable HCPsto take treatment decisions. For such software products, we would welcome a clear definition of medical device *monitoring* in updated guidance documents to facilitate their qualification.

 As confirmed by the FDASIA report,^12^ the development of software products used to take decisions with diagnosis or therapeutic purposes has increased in the last decades (called “decision support software in EU”^10^ or “CDS software in US”^5^). Unfortunately, no harmonized definition is available for CDS software. The authors of the viewpoint article came up with their own definition on CDS software: ‘*any software system that integrates personal patient data with external sources of medical knowledge to assist the HCPs in their decision-making process.’*^1^ Following MDCG guidance,^10^ decision support software is *intended to provide HCPs and/or users with recommendations for diagnosis, prognosis, monitoring and treatment of individual patients. *Following FDA guidance,^5^ CDS software would provide ‘*HCPs and patients with knowledge and person-specific information, intelligently filtered, or presented at appropriate times, to enhance health and healthcare*.’ Generally, the meaning of those definitions seems identical, however limitations exist in the practical qualification of CDS software as a medical device (SaMD). Van Laere et al^1^ are correctly requesting further guidance on how FDA will assess the availability of plain language description of the logic or rationale used by an algorithm and the availability of the elements forming the basis of the recommendations to the intended user. The understanding of the basis of a certain CDS might trigger that CDS software being exempted from the 21st Century Cures Act.^2^ This approach of a software recommendation being or not being understandable has not been considered in the EU MDCG guidance document as a criterion to qualify CDS SaMD. Additional clarification in guidance documents (US and EU) on the basis of the software recommendation and/or the action of the software on the data (eg, data analysis) would improve the qualification assessment of software products.

###  Is the Classification of Clinical Decision Support Software Different Between Jurisdictions? 

 The EU and FDA medical device legislations have leveraged the IMDRF risk-based framework for categorizing SaMD, based on the risk to patients if the software malfunctions.^13^ IMDRF’s categorization criteria and framework (Table 1)^13^ are not a regulatory classification, nor imply a convergence of classifications rules. Each jurisdiction has aligned her SaMD or medical device software (EU term) classification rules with this IMDRF framework in a unique way. As indicated by Van Laere et al, the EU MDR classification is much more stringent, especially due to the new classification Rule 11. Software intended to provide *information in the clinical management* is always classified as a Class IIa medical device in Europe, requiring the regulatory approval by a notified body (Table 2).^3,10^

**Table 1:** IMDRF SaMD Working Group - “Software as a Medical Device”: Possible Framework for Risk Categorization and Corresponding Considerations

**State of Healthcare Situation or Condition**	**Significance of Information Provided by SaMD to Healthcare Decision**		
	**Treat or Diagnose**	**Drive Clinical Management**	**Inform Clinical Management**
Critical	IV	III	II
Serious	III	II	I
Non-serious	II	I	I

Abbreviations: IMDRF, International Medical Device Regulators Forum; SaMD, Software as a Medical Device. Source: IMDRF SaMD Working Group.^13^

**Table 2:** MDCG 2019-11 Guidance on Qualification and Classification of Software in Regulation (EU) 2017/745 – MDR and Regulation (EU) 2017/746 – IVDR: Classification Guidance on Rule 11

**State of Healthcare Situation or Patient Condition**	**Significance of Information Provided by SaMD to Healthcare Decision Related to Diagnosis/Therapy**		
	**High** **Treat or Diagnose**	**Medium** **Drive Clinical Management**	**Low** **Inform Clinical Management**
Critical situation or patient condition	Class III	Class IIb	Class IIa
Serious situation or patient condition	Class IIb	Class IIa	Class IIa
Non-Serious situation or patient condition	Class IIa	Class IIa	Class IIa

Abbreviations: MDCG, Medical Device Coordination Group; MDR, Medical Device Regulation; SaMD, Software as a Medical Device; IVDR, In Vitro Diagnostic Regulation. Source: Medical Device Coordination Group Document MDCG 2019-11.^10^

 The FDA approach is more pragmatic. FDA has drafted guidance on CDS software functions with a specific focus on CDS software used for informing clinical management.^5^ The categorization method for regulatory oversight is based on the user (a HCP, patient, or caregiver) on the one hand and on the fact that the user can review the basis of the information on the other hand (Table 3). With this approach, FDA strikes the right balance between ensuring patient safety and promoting innovation by clarifying which products would be the focus of FDA’s oversight and which would not. As noted by Van Laere et al,^1^ two medical devices with the same intended use may obtain different classifications in those two jurisdictions. Unfortunately, EU MDR does not consider the lower significance of *informing clinical management* of the IMDRF framework nor the stage of healthcare condition of the patient, to allow a lower classification and thus regulatory burden for such CDS software (leading to a class IIa classification for low impact SaMD in non-serious situation or patient condition in Table 2).

**Table 3:** Draft Guidance on Clinical Decision Support Software: Summary of Regulatory Policy for CDS Software Functions

**IMDRF Risk Categorization**	**Can the User Independently Review the Basis?**^a^	**Intended User** **HCP**	**Intended User** **Patient or Caregiver**
		**FDA Regulation**	**FDA Regulation**
Inform and critical	Yes	Not a device	Oversight focus
	No	Oversight focus	Oversight focus
Inform and serious	Yes	Not a device	Oversight focus
	No	Oversight focus	Oversight focus
Inform and non-serious	Yes	Not a device	Enforcement discretion^b^
	No	Enforcement discretion^b^	Oversight focus

Abbreviations: FDA, Food and Drug Administration; IMDRF, International Medical Device Regulators Forum; HCP, healthcare professional; CDS, clinical decision support. ^a^ “Can the User Independently Review the Basis?” asks whether the function is intended for the purpose of enabling the user to independently review the basis for the recommendations so that it is not the intent that user relies primarily on any such recommendation. ^b^ “Enforcement Discretion” indicates that, based on our current understanding of the risks of these devices, does not intend at this time to enforce compliance with applicable device requirements. Source: US FDA.^5^

###  Are There Other Limitations in the Practical Implementation of Medical Device Legislations?

 Advanced technologies such as AI are becoming more frequently used in healthcare for diagnostic or therapeutic purposes. The US FDA clearly has taken the path forward for embracing this technology by drafting an AI/machine learning SaMD action plan.^14^ In Europe, guidance on interpretation of significant changes to AI are not yet available. Due to the promotion of those innovative products to the market, there is a growing need for software experts in Europe, especially within the conformity assessment bodies for reviewing the CE certification applications. As suggested by the FDA,^14^ there is a great need for more harmonization in the AI domain, more specifically in the development of good machine learning practice through the creation of consensus standards and other guidelines.

## Conclusion

 The topic of CDS software is relevant as it became more widespread in different healthcare fields. With the evolution of software technologies such as AI, CDS software is expected to consolidate its position in healthcare. To keep up with the technological evolutions, it is desirable that the different regulators consult with industry and users and publish and continuously update more practical guidance documents. US FDA already takes a proactive approach in providing multiple guidance documents^4-9^ on software, but also on CDS software and an action plan for AI/machine learning based SaMD.^14^ Although the European Commission had good intentions when considering the IMDRF risk categorization framework, it did not classify CDS software which informs HCPs for non-serious patient conditions in a low medical device class. This results to the fact that manufacturers will most likely need a notified body to place this type of products on the market in Europe. This will certainly increase the costs and timelines to go to the market, probably making Europe a less attractive market to deploy digital health innovations in a first stage. Currently, some manufacturers are reducing their product portfolio in Europe to limit regulatory cost related to EU MDR. As expected by Van Laere et al,^1^ the real clinical impact on patient’s health will only be measurable in the next few years.

## Ethical issues

 Not applicable.

## Competing interests

 Authors declare that they have no competing interests.
